# Comparative assessment of plant protection products: how many cases will regulatory authorities have to answer?

**DOI:** 10.1186/s12302-014-0011-8

**Published:** 2014-06-28

**Authors:** Michael Faust, Carolina Vogs, Stefanie Rotter, Janina Wöltjen, Andreas Höllrigl-Rosta, Thomas Backhaus, Rolf Altenburger

**Affiliations:** 1 Faust & Backhaus Environmental Consulting, Fahrenheitstr. 1, Bremen, 28359 Germany; 2 Department Bioanalytical Ecotoxicology, UFZ - Helmholtz Centre for Environmental Research, Permoser Str. 15, Leipzig, 04318 Germany; 3 Federal Environment Agency, Wörlitzer Platz 1, Dessau-Roßlau, 06844 Germany

**Keywords:** Substitution principle, Comparative risk assessment, Pesticides, Plant protection products, Active substances, Candidates for substitution, PBT

## Abstract

**Background:**

The substitution principle has been included in the EU pesticides legislation as a new element. Comparative assessments will have to be conducted for all uses of plant protection products (PPPs) that contain active substances with certain hazardous properties, the so-called candidates for substitution (CFS). This study investigated the resulting workload in terms of the number of cases for comparative assessments that regulatory authorities may have to face. The analysis was carried out for Germany as an example.

**Main text:**

In Germany, the requirement for comparative assessments may affect up to 25% of all PPPs and around 50% of all uses of PPPs. In absolute terms, these are around 350 candidate products with 1,850 different uses. Alternative products without CFS may be available for around 40% of these uses. On average, a candidate product is authorised for around 18 different uses. For 11 of these uses, no alternatives are authorised. For the remaining seven uses, slightly more than seven alternatives are available on average. Multiplication of these factors gives an indicative figure of around 18,500 possible pairwise comparisons of candidate products with alternative products for every common use.

**Conclusions:**

The high number of expectable cases poses a formidable challenge for the efficient conduct of the new task of comparative assessments by competent Member States authorities. To this end, new data handling systems, assessment procedures, and decision rules need to be established.

## Background

### The substitution principle in the EU pesticides legislation

The substitution principle is a new element of the legislation on plant protection products (PPPs) in the European Union (EU). It was introduced with the new Regulation (EC) No 1107/2009 [1], in the following shortly denoted as the PPP Regulation. This replaced the old Directive 91/414/EEC on PPPs [2] in June 2011. In parallel, the substitution principle was also included in the new Regulation (EU) No 528/2012 on biocidal products [3], which came into force in September 2013. PPPs and biocidal products are collectively denoted as ‘pesticides’ under EU law, as has been defined in Article 3 of Directive 2009/128/EC on the sustainable use of pesticides in the European Community (EC) [4]. As a common rule, pesticides shall not be placed on the market or used unless they have been authorised in accordance with the applicable regulations. In general, ‘substitution’ of pesticides means that an authorisation is refused or withdrawn in favour of an alternative product or a non-chemical control or prevention method which presents a ‘significantly lower risk’, according to Annex IV of the PPP Regulation (EC) No 1107/2009 and Article 23 of the biocidal products Regulation (EU) No 528/2012, respectively. In detail, the conditions, rules, and criteria for applying the substitution principle differ for PPPs and for biocidal products. In this paper, we focus on substitution under the Regulation for PPPs.

The inclusion of the substitution aspect in the EU pesticides legislation is an outcome of a broader and long-lasting discussion about the guiding principles of chemicals regulation under EU law. As a generic policy principle, substitution means the replacement of hazardous chemical substances and products by less hazardous alternatives [5]. Whether this idea should be established as a legal demand for actors in the field has been subject to heated debates. Opponents, such as the German chemical industries for instance, argued that substitution was superfluous if safe use of a hazardous chemical could be ensured by appropriate risk management measures [6]. In 2001, during the preparation of the REACH legislation, the Commission of the European Communities (COM) considered the substitution of hazardous chemicals as one of the ‘key elements’ of the proposed ‘Strategy for a future Chemicals Policy’ [7]. Five years later, in the final REACH legislation [8], legal requirements for feasibility analyses for substitution were, however, confined to substances of very high concern (SVHC) that are subject to authorisation (Article 55 of Regulation (EC) No 1907/2006). In all three pieces of legislation, where substitution has now been included as an element of authorisation procedures (REACH, biocidal products and PPPs), hazardous properties of chemicals serve only as a trigger for considerations for substitution, but are considered insufficient for decision making. Instead, comparative risk assessments of products have to be conducted as the basis for substitution decisions, which is novel and challenging.

Conventional risk assessments for individual PPPs, as they have been established under the old Directive 91/414/EEC, aim to ensure that regulatory acceptable exposure levels are not exceeded, but they do not provide incentives for reducing risks any further. This is changed by the complementary instrument of comparative risk assessment which supports a process of continuous improvement by identifying those PPPs that allow to achieve a desired purpose with minimal risks at a given point in time. This is particularly favourable for environmental risks, where the authorisation requirements still allow to tolerate temporary adverse effects as acceptable. Moreover, acceptable exposure levels for many pesticides on the market are only achievable by applying risk mitigation measures, such as protective equipment for workers or buffer zones between sprayed agricultural land and surface waters. Such measures may fail accidentally or may be disregarded negligently. Substitution of such products by alternatives that require less risk mitigation measures is therefore desirable and shall be supported by the new instrument of comparative assessments.

While the intended improvements are clear, the detailed procedures and methodologies for applying the substitution principle are not. Only in the Nordic countries, particularly in Sweden, the principle has been included in the national chemicals legislation since the beginning of the 1990s [9]. Other EU Member States (MS) have no comparable legislative tradition. Against this background, there is high uncertainty about potential impacts of this new element of EU pesticides legislation and the best way towards its efficient implementation.

### Candidates for substitution

Plant protection products contain one or more active substances. Under EU law, PPPs are authorised on the Member States level, while active substances are approved on the Community level. Approved active substances are included in a positive list established by the European Commission. Member States shall not authorise PPPs that contain active substances other than those on the positive list. Authorisations are only granted for specified uses, usually defined by a combination of a protected crop and a targeted pest.

The revised legislation now requires that certain active substances shall be approved by the European Commission only as ‘candidates for substitution’ (CFS) and listed separately from other approved active substances. Member States shall not grant authorisation to PPPs that contain such CFS, if a comparative assessment reveals that a significantly safer alternative is available for the same use.

CFS are active substances that have one or more of the hazardous properties listed in Table 1. As laid down in the PPP Regulation, their identification constitutes one task within the regular assessment of active substances on Community level. In order to speed up the process for the already approved active substances, an obligation for the European Commission (COM) was included in the PPP Regulation to establish an initial list of CFS until the end of 2013. However, completion of this task is now expected to be delayed by a few months [10]. At the time of writing of this manuscript (March 2014), the official list was not yet available.

**Table 1:** **Criteria for the identification of active substances as candidates for substitution (CFS)**

**Number**	**Legal text (Regulation (EC) No 1107/2009, Annex II, point 4)** **[** 1 **]** ^**a**^
1	Its ADI, ARfD, or AOEL is significantly lower than those of the majority of the approved active substances within groups of substances/use categories
2	It meets two of the criteria to be considered as a PBT substance
3	There are reasons for concern linked to the nature of the critical effects (such as developmental neurotoxic or immunotoxic effects) which, in combination with the use/exposure patterns, amount to situations of use that could still cause concern, for example, high potential of risk to groundwater; even with very restrictive risk management measures (such as extensive personal protective equipment or very large buffer zones)
4	It contains a significant proportion of non-active isomers
5	It is or is to be classified, in accordance with the provisions of Regulation (EC) No 1272/2008, as carcinogen category 1A or 1B, if the substance has not been excluded in accordance with the criteria laid down in point 3.6.3^b^
6	It is or is to be classified, in accordance with the provisions of Regulation (EC) No 1272/2008, as toxic for reproduction category 1A or 1B if the substance has not been excluded in accordance with the criteria laid down in point 3.6.4^b^
7	If, on the basis of the assessment of Community or internationally agreed test guidelines or other available data and information, reviewed by the Authority, it is considered to have endocrine disrupting properties that may cause adverse effects in humans if the substance has not been excluded in accordance with the criteria laid down in point 3.6.5^b,c^

^a^The criteria apply independently, i.e. CFS meet one or more of them. ^b^Points 3.6.2 to 3.6.5 of Annex II of Regulation (EC) No 1107/2009 [1] define hazard-based criteria for substances that must not be approved, so-called cut-off criteria. ^c^For endocrine disrupters, currently the interim criteria laid down under point 3.6.5 of Annex II of Regulation (EC) No 1107/2009 [1] apply, i.e. substances classified as carcinogenic category 2 and toxic for reproduction category 2.

As a support for the preparation of the initial list, COM commissioned a contract study to the Food Chain Evaluation Consortium (FCEC). The FCEC delivered their study report in July 2013 [11]. Subsequently, the report was presented to the competent authorities of the Member States, and it was made available to all stakeholders via the Commission's CIRCA platform [12].Thus, although not formerly published, the report is in the public domain and was accessible for the purposes of this paper via the German Federal Environment Agency (UBA).

It was not the task of the FCEC to set up the initial list of CFS - this is the privilege of COM - but to do the necessary preparatory work, which was

(i) to compile the data needed for decision-making from the legally relevant documents, i.e. those documents on which the original decisions for approval of active substances have been based, such as the official Review Reports, EFSA Conclusions, and Draft Assessment Reports, and
(ii) to explore options for the interpretation and operationalisation of the criteria for the identification of CFS (Table 1), where the legal text and the available data leave room for judgments and where corresponding rules for data assessments had not already been fixed in a corresponding Commission Working Document on ‘Evidence Needed to Identify POP, PBT and vPvB Properties of Pesticides’ [13].

As a consequence, the FCEC report did not directly provide a list of CFS, but it included separate lists of active substances that were considered to fulfil individual CFS criteria or sub-criteria, for example persistence in soil, in sediments, and in water. Where applicable, the report provided various versions of these lists, each representing the outcome of a different interpretation of a legal criterion, such as different measures and trigger values for a ‘significantly lower ADI’. In each of these cases the report provided arguments for the option that the authors considered to be the most appropriate one.

Thus, by combining the information from these individual lists, it is possible to obtain a list of potential CFS that have a high chance for becoming actually included in the initial list of CFS that COM is going to establish. This opportunity was used for the purpose of this study.

### Comparative assessments

In the future, EU Member States shall perform a comparative assessment whenever they evaluate any application for authorisation of a PPP that contains a CFS, in the following shortly denoted as candidate product. A comparative assessment may be initiated by an application for the authorisation of a new candidate product, for the renewal of an existing authorisation, or for the amendment of an authorisation for new uses of a candidate product. Comparative assessments must be performed for each use of a candidate product. A candidate product shall not be authorised for a use for which an alternative chemical product or a non-chemical control method is available, if the following requirements are fulfilled (Article 50 in conjunction with Annex IV of the PPP Regulation):

(i) Experience from practical use of the alternative is available.

(ii) The alternative has a comparable efficacy against target pests.

(iii) The alternative can be used without significant economic or practical disadvantages, including impacts on the so-called minor uses.

(iv) The substitution does not compromise resistance management and the minimisation of the occurrence of resistance.

(v) The alternative product or method is ‘significantly safer for human or animal health or the environment’.

Thus, the comparative assessment can be divided into two major parts: a comparative agronomic assessment covering points (i) to (iv) and a comparative safety assessment as required by point (v). This paper focuses on the safety assessment part. In addition, it does not discuss comparisons of chemical PPPs with non-chemical protection methods (e.g. mechanical methods or bio-pesticides such as viruses and bacterial strains).

Annex IV to the PPP Regulation clarifies that the increase in safety that is achieved by a substitution shall be demonstrated in terms of a ‘significantly lower risk’. In general, competent authorities shall identify such significant differences in risk ‘on a case-by-case basis’. A significance level is not specified for the comparative human health risk assessment but for the environmental risk assessment: ‘if relevant, a factor of at least 10 for the toxicity/exposure ratio (TER) of different plant protection products is considered a significant difference in risk’ [1].

For conducting the agronomic part of the comparative assessments, guidance has been developed by EPPO, the European and Mediterranean Plant Protection Organization [14]. For the risk assessment part, COM is currently working on a guidance document, based on a proposal by Sweden [15]. This guidance aims to support the Member States, but it will not establish detailed and legally binding rules. Basically, it will be up to the decision of the Member States on how they actually conduct comparative risk assessments.

### Regulatory impact

In order to prepare for the new task of conducting comparative product assessments, competent authorities need to obtain an overview about the dimension of the additional workload they may be confronted with. Earlier assessments of the potential impacts of the new PPP legislation estimated that about 15% to 25% of the approved active substances could become candidates for substitution [16,17], but these studies were based on limited and uncertain databases and they did not further explore the consequences in terms of numbers of potentially affected products and uses of products, and in terms of numbers of pairs or groups of products that could become subject to a comparative risk assessment. However, with the data from the recent FCEC report, a solid basis is now available for such calculations.

### Aims and approach

This study aimed to estimate the potential number of cases for which a comparative assessment will need to be carried out, in terms of (i) the number of CFS-containing candidate products, (ii) the number of different uses of such candidate products, (iii) the number of uses of candidate products for which alternative products are available, and (iv) the possible number of pairwise comparisons of candidate and alternative products for all common uses. For the purpose of this study, we assumed that candidate products are only compared to alternative products that do not contain any CFS. In principle, neither the legal text nor the available draft of the EU Guidance Document excludes the possibility of a substitution of a candidate product by another candidate product, if a significant risk reduction would result. However, we assumed that this would be a rare situation and excluded it from further consideration.

The exercise did not cover the whole European Union but was conducted for the German PPP market as an example. The calculations were based on the simplistic assumption of a static market share of candidate products. This means that authorisations for all uses of all CFS-containing PPPs that are currently on the market would become subject to renewal in the future or that they will be replaced by an equal number of new candidate products with an equal number of uses. In this way we assumed to obtain upper limit estimates of the potential number of cases for comparative assessments that may accumulate over a period of time equal to the average duration of authorisations (typically granted for 10 years).

The identification of CFS results from hazardous properties for humans or for the environment or for both. For human and environmental risk assessments of PPPs, different aims, methods, and procedures apply and they are often performed separately by different institutions. In Germany, for instance, the Federal Institute for Risk Assessment (BfR), the Julius Kühn Institute (JKI), and the Federal Environment Agency (UBA) separately assess risks for humans, for honey bees, and for the environment, respectively. Therefore, we were also interested to see whether the number of cases for comparative risk assessments would be significantly reduced, if comparative environmental assessments would be conducted only for those candidate products that contain CFS as identified by hazardous environmental properties. To this end, we performed all calculations twice: once for all candidate products and once only for those products that contain CFS that have been identified for reasons of environmental hazards (exclusively or in addition to human health hazards). The way by which we achieved this discrimination is explained in the ‘Main text’ section.

The list of potential CFS resulting from the FCEC report is currently subject to final revisions by the Commission and the Member States. In order to avoid any false discrimination of substances or products in this interim situation, this paper is exclusively focused on the quantitative aspects of the subject, providing numbers of substances, products, and uses, but no names. As a result of the ongoing revisions, the forthcoming official CFS list can be expected to be slightly but not substantially shorter than assumed in this paper [10]. Consequently, the figures provided in this paper for the number of candidate products can be expected to represent a slight overestimation of the findings that will be obtainable by performing the same analyses on the basis of the final CFS list.

## Main text

### How many CFS?

Almost 100 active substances are potential candidates for substitution, which is roughly a quarter of all approved active substances. This derives from an examination of the information provided in the FCEC report to the Commission [11], as detailed in the ‘Methods’ section. The number refers to the list of approved active substances laid down in the Annex to Commission Implementing Regulation (EU) No 540/2011 [18], as amended until 31 January 2013. Where the legal definition of an approved active substance includes different varieties of a parent compound or where the approval applies to a defined group of compounds, these were counted as a single entity. Earlier impact studies expected CFS proportions between 15% and 25% [16,17]. Our findings show that these prognoses indicated the dimension of the problem quite accurately.

Potential CFS are spread across all major use categories, as defined in the EU Pesticides database [19]. Between 10% and 25% of substances used as fungicides, insecticides, plant growth regulators, or multi-purpose pesticides are potential CFS (Table 2). For acaricides and herbicides, the proportions are higher, with around 40% of the substances being potentially affected. From the small group of rodenticides, even 60% are potential CFS.

**Table 2:** **Proportion of active substances identified as potential CFS, broken down by use categories**

**Use category** ^**a**^	**Total number of approved active substances** ^**b**^	**Proportion of potential CFS** ^**c**^ **(%)**
AC	8	38
FU	103	25
HB	110	41
IN	55	18
PG	25	12
RE	17	0
RO	5	60
Other	14	0
Multi	35	23
Not assigned	6	0
All	378	26

^a^Assignment to use categories as given in the EU Pesticides database [19]; AC, acaricides; FU, fungicides; HB, herbicides; IN, insecticides; PG, plant growth regulators; RE, repellents; RO, rodenticides; Other, attractants, bactericides, elicitors, molluscicides, nematicides, and plant activators; Multi, multiple use categories apply to the same substance, also including uses as dessicant in addition to one or more of the other listed categories. ^b^Reference date: 31 January 2013; counting of approved active substances refers to the legal definitions listed in Commission Implementing Regulation (EU) 540/2011 [18]; from a chemical perspective, parts of these are groups of substances or mixtures of substances; in addition to chemicals, the legal substance definition also includes viruses and bacteria, the so-called bio-pesticides. ^c^Percentages rounded to integer values; potential CFS are those identified in the FCEC report [11], as detailed in the text.

The so-called PBT properties have an outstanding importance for the number of substances identified as a potential CFS (Table 3). Almost 80% of the potential CFS fulfil the second CFS criterion (see Table 1), which means that they meet two of the criteria to be considered as persistent (P), bioaccumulative (B), and/or toxic (T), as defined under point 3.7.2 of Annex II to the PPP Regulation. Low ADI, ARfD, or AOEL values; reproductive toxicity class 1A or 1B; or endocrine disrupting properties (criteria 1, 6, and 7 in Table 1) have a lower impact on the number of potential CFS, i.e. 22%, 9%, and 7% of the candidates, respectively. Even less influence on the number of CFS has criterion 4 (Table 1), i.e. a significant proportion of non-active isomers: only 2% of the potential CFS meet this criterion. The remaining two criteria 3 and 5 (‘nature of critical effects’ and carcinogens class 1A/1B) were not found to apply to any approved active substances in the FCEC report. Sixteen percent of all potential CFS were found to meet two or more of the seven identification criteria. There is no obvious association between applicable CFS criteria and the use categories of substances (Table 3). Substances meeting two PBT criteria, for instance, come from all major use categories, whereupon the proportions basically reflect the different sizes of the use groups.

**Table 3:** **Breakdown of the number of potential CFS by use categories and identification criteria**

**CFS criterion** ^**a**^		**Use category** ^**e**^							**Sum**
		**AC**	**FU**	**HB**	**IN**	**PG**	**RO**	**Multi**	
1. Low ADI/ARfD/AOEL		0	6	8	2	1	0	5	22
2. Two PBT criteria	T_AQUA_^b^	3	20	32	8	2	2	3	70
	T_HUMAN_^c^	0	8	8	1	0	0	3	20
	All	3	23	35	8	2	2	4	77
3. Nature of critical effects		0	0	0	0	0	0	0	0
4. Non-active isomers		0	1	1	0	0	0	0	2
5. Carcinogen 1A/1B		0	0	0	0	0	0	0	0
6. Toxic for reproduction 1A/1B		0	3	5	0	0	1	0	9
7. Endocrine disrupting properties^d^		0	2	4	1	0	0	0	7
All		3	26	45	10	3	3	8	98

^a^The numbering refers to the full legal definition of criteria as given in Table 1; corresponding short descriptions are those used in the EU pesticide database [19]; different criteria for identification as a CFS may apply to one and the same substance; therefore figures in lines ‘All’ do not equal sums of values given in the columns. ^b^T_AQUA_: substances fulfil the toxicity criterion for water organisms laid down under point 3.7.2.3 of Annex II to Regulation (EC) No 1107/2009 [1], in addition to the P or the B criterion. ^c^T_HUMAN_: substances fulfil any of the human toxicity criteria defined under point 3.7.2.3 of Annex II to Regulation (EC) No 1107/2009 [1] in terms of CMR or STOT RE classifications, in addition to the P or the B criterion. ^d^For endocrine disrupters, the interim criteria laid down under point 3.6.5 of Annex II of Regulation (EC) No 1107/2009 [1] were applied, i.e. substances classified as carcinogenic category 2 and toxic for reproduction category 2. ^e^Assignment to use categories as given in the EU Pesticides database [19]; AC, acaricides; FU, fungicides; HB, herbicides; IN, insecticides; PG, plant growth regulators; RO, rodenticides; Multi, multiple use categories apply, also including uses as dessicant, nematicide, or repellent in addition to one or more of the categories AC, FU, HB, IN, PG, and RO.

The seven legal CFS criteria include both human health hazards and environmental hazards, but both aspects are separable, as explained in the following. CFS criteria 1, 5, 6, and 7 (low ADI/ARfD/AOEL, carcinogenicity, reproductive toxicity, and endocrine disrupting properties that may cause adverse effects in humans as defined in the legislation) are exclusively based on tests used for human toxicity assessments. Criterion 3 (nature of critical effects) could be interpreted to include both aspects but was not found to be relevant for the initial list of CFS. Criterion 4 (non-active isomers) clearly has a relevance for both human and environmental hazard and risk assessments. Criterion 2 (two PBT criteria) theoretically comprises three different situations: P + B, P + T, and B + T. Practically, however, the combination P + B has apparently no relevance for the initial CFS list. Hence, criterion 2 always combines an exposure indicator (P or B) with a toxicity indicator (T). As laid down under point 3.7.2.3 of Annex II of the PPP Regulation, two different types of toxicity indicators are applicable: indicators of human toxicity (CMR and STOT RE classifications) and indicators of aquatic toxicity (long-term NOEC), in the following denoted as T_HUMAN_ and T_AQUA_, respectively. As a consequence, it is possible to distinguish between CFS that meet criterion 2 for reasons of human health protection (T_HUMAN_ in combination with P or B) or for reasons of environmental protection (T_AQUA_ in combination with P or B), or both. In summary of these considerations, CFS identified by environmental hazard criteria are those that meet the T criterion for water organisms in addition to the P or the B criterion, and those that contain a significant proportion of non-active isomers. All other CFS are identified for reasons of human health hazards.

By applying these categorisations, we found that only 19% of the potential CFS are exclusively identified by human health criteria; 81% meet environmental hazard criteria, whereby 27% meet both human and environmental hazard criteria and 54% are exclusively identified for reason of environmental hazards.

### How many candidate products?

The analysis of the German register of authorised PPPs revealed that 25% of the products contain one or more potential CFS and may hence be considered as potential candidate products (Table 4). This proportion is almost identical to the share of CFS in the number of approved active substances. Interestingly, however, only 67 out of 98 potential CFS were actually found in any PPP on the German market, i.e. around 30% of the CFS currently have no relevance for comparative assessments in Germany. In absolute figures, 351 out of 1,378 authorised PPPs are potential candidate products. Two hundred thirty-seven products contain a potential CFS that has been identified for reason of environmental hazards, which is a share of 17% of all products and 67% of all potential candidate products. Thus, on the level of products on the German market, the importance of environmental hazard criteria for the number of candidates is only slightly lower than seen on the level of active substances, where the corresponding fraction is 81% of all potential CFS.

**Table 4:** **Proportion of candidate products containing potential CFS authorised in Germany**

**Use category** ^**a**^	**Total number of authorised PPPs** ^**b**^	**Proportion of candidate PPPs containing one or more potential CFS** ^**c**^	
		**Containing any potential CFS (%)**	**Containing CFS identified by environmental hazard criteria** ^**d**^ **(%)**
Herbicides	567	25	21
Fungicides	308	45	32
Insecticides	281	25	6
Acaricides	109	12	2
Plant growth regulators	59	14	7
Molluscicides	58	0	0
Rodenticides	41	2	2
Glue, sealing wax	31	0	0
Repellents	29	0	0
Sprout inhibitors	18	0	0
Bactericides	9	11	11
Pheromones	3	0	0
Viricides	1	0	0
Nematicides	1	0	0
All^b^	1,378	25	17

As of May 2013. ^a^Categorisation as given in the BVL database on plant protection products [20]; this categorisation of products is largely but not entirely consistent with the categorisation of active substances used in the EU pesticide database [19] (see preceding tables). ^b^The given categorisation of products is non-exclusive, i.e. multiple use categories may apply to a single product; this is the case for around 10% of all PPP; as a consequence, the overall number of 1,378 PPPs is smaller than the sum of values in the column. ^c^Percentages rounded to integer values; potential CFS are those identified in the FCEC report [11], as detailed in the text. ^d^Active substances that fulfil the toxicity criterion for water organisms laid down under point 3.7.2.3 of Annex II to Regulation (EC) No 1107/2009 in addition to the P or the B criterion as defined under points 3.7.2.1 and 3.7.2.1 of the same Annex II [1], and active substances that contain a significant proportion of non-active isomers; human health criteria may apply additionally.

The potential candidate PPPs are spread across seven major use categories, with shares ranging between 2% and 45%. This is essentially the same situation as observed on the level of CFS. Interestingly, however, the proportions can be quite different for the same use category. In the small group of rodenticides, for instance, 60% of the active substances were found to be potential CFS, but no more than 2% of the rodenticidal products authorised in Germany actually contain any of these CFS. The opposite situation does also occur: In the large group of fungicides, for instance, 25% of the active substances are potential CFS, but 45% of the products on the German market contain one or more of these CFS. The high proportion of 45% potential candidate products amongst fungicides is exceptional; for all other use categories, the fractions are at or below the average of 25%.

### How many uses of candidate products?

On average, potential candidate products are authorised for a broader spectrum of uses than other PPPs. As a consequence, they may necessitate comparative assessments for a disproportionally high number of uses; they account for no more than 25% of all products (see above), but they are authorised for around 50% of all uses. This is the essential outcome of an analysis detailed in the following.

Authorised uses of PPPs are defined in terms of a combination of a crop and a pest from which the crop shall be protected. A small number of other treatment objects (such as food storage rooms) and treatment aims (such as plant growth regulation) are subsumed here also under the terms crop and pest, respectively. Using the status of May 2013, a total of 1,378 PPPs was authorised in Germany for a total of 3,606 uses, defined as different combinations from of a total of 309 crops and 477 pests (Table 5). The fraction of 351 potential candidate products (25% of all products) was authorised for a total of 1,863 uses (52% of all uses), defined as different combinations from 209 crops (68% of all crops) and 264 pests (55% of all pests). The sub-fraction of 237 potential candidate products that contain CFS identified by environmental hazard criteria (17% of all products) was authorised for a total of 1,501 uses (42% of all uses), defined as different combinations from 186 crops (60% of all crops) and 228 pests (48% of all pests).

**Table 5:** **Number of plant protection products, protected crops, controlled pests, and authorised uses in Germany**

**Parameter**			**All PPPs**	**Candidate PPPs containing one or more potential CFS**	
				**Containing any potential CFS**	**Containing CFS identified by environmental hazard criteria** ^**a**^
Products	Total number		1,378	351	237
Crops^b^	Total number of different crops for which use of PPPs has been authorised		309	209	186
	Number of different crops for which use of an individual PPP has been authorised	Min	1	1	1
		Max	85	85	85
		Median	4	4	4
		Mean^e^	7	7	7
Pests^c^	Total number of different pests against which use of PPPs has been authorised		477	264	228
	Number of different pests against which use of an individual PPP has been authorised	Min	1	1	1
		Max	64	39	39
		Median	3	4	4
		Mean^e^	5	5	5
Uses^d^	Total number of different uses for which PPPs have been authorised		3,606	1,863	1,501
	Number of different uses for which an individual PPP has been authorised	Min	1	1	1
		Max	337	337	337
		Median	6	8	9
		Mean^e^	13	18	18

As of May 2013. Broken down by potential CFS content of products. ^a^As defined in the corresponding footnote to Table 4. ^b^Including other authorised treatment objects such as food storage rooms for instance. ^c^Including other authorised treatment aims such plant growth regulation for instance. ^d^Defined by a combination of a crop (or another treatment object) and a pest from which the crop shall be protected (or another treatment aim). ^e^Arithmetic mean rounded to integer values.

On average, every PPP is authorised for use against five different pests in seven different crops. For potential candidate products, the same mean values apply. On the level of uses, however, the situation is different: while the mean number of authorised uses is 13 for all PPPs, potential candidate products have an average of 18 different authorised uses.

### How many alternatives are available?

For 767 different uses of potential candidate products, at least one CFS-free alternative product is authorised (Table 6). This is slightly more than 40% of all 1,863 uses of potential candidate products. For around 9%, even more than 10 different alternative products are authorised. For the sub-fraction of potential candidate products that contain CFS identified by environmental hazard criteria, the corresponding proportions are slightly higher: at least one alternative is available for 687 of 1,501 uses, i.e. almost 46%. For almost 12%, even more than 10 different alternative products are authorised.

**Table 6:** **Potential availability of alternative products for authorised uses of CFS-containing candidate products in Germany**

**Parameter**	**All potential candidate products (** ***n*** **= 351)**	**Potential candidate products containing CFS identified by environmental hazard criteria** ^**a**^ **(** ***n*** **= 237)**
Availability of alternative products for uses of candidate products	Number of uses	
No alternatives available	1,096	813
Any alternatives available	767	687
1 alternative available	220	162
2 alternatives available	126	112
3 to 5 alternatives available	172	163
6 to 10 alternatives available	82	77
11 to 20 alternatives available	112	118
21 to 50 alternatives available	42	44
51 to 100 alternatives available	12	10
More than 100 alternatives available	1	1
Average spectrum of uses of candidate products	Mean number of uses^b^	
Including all uses of candidate products	18	18
Including only uses of candidate products for which alternatives are available	7	8
Average availability of alternatives	Mean number of alternative products^b^	
For all uses of candidate products	3	4
For all uses of candidate products for which alternatives are available	7	8

Scenario based on the status of authorised products in May 2013. Candidate products are products containing one or more potential CFS; alternatives are products containing no potential CFS. ^a^As defined in the corresponding footnote to Table 4. ^b^Arithmetic mean rounded to integer values.

On average, one or more alternative products are authorised for around 7 out of 18 different uses of a potential candidate product. For each of these seven uses, the mean number of available alternative products is also around seven. For the sub-fraction of potential candidate products that contain CFS identified by environmental hazard criteria, these mean values differ only marginally.

### How many cases for comparative assessments?

In principle, every application for authorisation of a candidate product constitutes a case for comparative assessments, as required by the PPP Regulation. While the assessments may be stopped in an early phase, if alternatives are not available for any of the uses of the candidate product, our analysis revealed that only for 6 out of 351 potential candidate products no alternative products are authorised for any of their uses. The remaining 345 potential candidate products (around 98%) may trigger more detailed comparative assessments, at least of the agronomic aspects, and where appropriate also of the safety aspects. For the sub-fraction of potential candidate products that contain CFS identified by environmental hazard criteria, the proportion is basically the same (Table 7).

**Table 7:** **Potential number of cases for comparative risk assessment of plant protection products in Germany**

**Cases defined in terms of…**		**Counts**	
		**For products containing any potential CFS**	**For products containing CFS identified by environmental hazard criteria** ^**a**^
Products^b^	Number of all candidate products	351	237
	Number of candidate products for which alternatives are available for one or more of their uses	345	232
Uses	Number of all authorised uses of candidate products	1,863	1,501
	Number of authorised uses of candidate products for which alternative products are available	767	687
Products × Uses	Number of all candidate products times average number of uses^c^	6,232	4,175
	Number of all candidate products times average number of uses for which alternatives are potentially available^c^	2,569	1,910
Products × Uses × Alternatives	Number of all possible pairwise risk comparisons of candidate products with alternative products for all common uses^d^	18,479	15,287

Scenario based on the status of authorised products in May 2013. ^a^As defined in the corresponding footnote to Table 4. ^b^Candidate products are products containing one or more potential CFS; alternatives are products containing no potential CFS. ^c^Calculated with non-rounded values for mean numbers of uses. ^d^Calculated by multiplying the number of all candidate products with the average number of uses for which alternatives are potentially available and the mean number of such alternatives for every use; non-rounded mean values were used for the calculations.

In terms of affected uses, more or less detailed comparative assessments may be required for the 767 different uses of potential candidate products for which at least one alternative product is currently authorised. However, even for the same use, not all candidate products will become subject to authorisation or re-authorisation at the same time. Whenever an authorisation is requested for a single candidate product, comparative assessments will have to be conducted for all its uses. This means that comparative assessments for the same use may have to be conducted repeatedly over time. Therefore, as an additional indicator of the potential workload for competent authorities, the number of potential candidate products can be multiplied with the average number of uses for which alternatives are potentially available. This yields an indicative figure of roughly 2,500 cases of use-specific assessments of candidate products that may become necessary during the next years. For the sub-fraction of potential candidate products that contain CFS identified by environmental hazard criteria, the figure is roughly 25% smaller.

It must be noted that our analysis of uses was based on the exact wording of the use descriptions in the German PPP register. Hence, we did not explore the potential for substituting a candidate product that is only used for a specific pest and/or crop by an alternative product that is authorised for broad-spectrum use against a large group of pests and/or in multiple crops. This could further increase the number of comparative product assessments.

In addition to the number of uses, the number of available alternative products must be taken into account. The broader the spectrum, the higher will be the workload for comparatively assessing all products that are available for a given use. As a further workload indicator, we therefore derived the product of all three factors: The number of potential candidate products × the average number of uses for which alternatives are potentially available × the average number of alternative products that is available for each of these uses. This results in roughly 18,500 cases. This figure is the number of all possible pairwise comparisons of candidate products with alternative products for all common uses of two products. For the sub-fraction of potential candidate products that contain CFS identified by environmental hazard criteria, the figure is roughly 20% smaller.

## Discussion

Estimated case numbers in this paper are based on a draft CFS list and on a simplistic static scenario, reflecting neither fluctuations in the number of authorised products nor any potential future trends. Therefore, the figures should not be taken as precise estimates but rather as indications of the expectable dimension of the upcoming demands on comparative assessments. In any case, the results of the present study indicate that several thousands of cases might need to be evaluated. Hence, the new task of performing comparative assessments of PPPs may pose a formidable challenge to competent authorities. Not only are the quantitative demands considerable, the established assessment methodologies for PPPs and the existing organisational arrangements are also only designed for checking compliance of individual PPPs with legal acceptability criteria. They were not developed for comparing products with each other. New data handling systems, assessment procedures, and decision rules might hence be required for efficiently dealing with the new task.

The comparative agronomic assessment and the comparative safety assessment may be performed either subsequently or in parallel. The EPPO guidance on comparative assessment proposed that the agronomic assessment should be carried out first [14], and the latest draft of the forthcoming Commission guidance document suggests the same [15]. With the aim of optimal efficiency of the overall procedure, this appears to be a rational and self-evident approach, as the agronomic assessment can be expected to provide a strong filter. Thus, the novel and demanding task of comparing the human and environmental risks of different products would be limited to the lowest possible number of cases. On the other hand, however, it must be taken into consideration that the agronomic part of the assessment includes criteria that need further interpretation and specification, in particular the requirement for no ‘significant’ economic or practical disadvantages. Corresponding definitions of significance may be based on isolated agronomic considerations, but it is also imaginable, that the assessment of the significance of economic disadvantages should take account of potential advantages for human health and the environment. Thus, the number of cases for comparative human and environmental risk assessments will depend on the detailed criteria and procedural arrangements of the overall process.

The workload for performing a complete set of comparative human and environmental risk assessments for all the uses of a candidate product will certainly be quite different, depending on the respective product. These different assessment situations result from the different numbers of uses of potential candidate products, which vary between one and a few hundreds, and also from the different numbers of potentially available alternatives, which range between zero and more than a hundred (Tables 5 and 6). In addition, the initial comparative assessments of substances available for a certain common use may be more demanding than a repeated assessment that is triggered by an application for authorisation of another product for the same use. Furthermore, the risks resulting from different uses of the same product may be quite similar or even identical, and most likely there will also be situations where the available spectrum of candidate products and alternative products is identical for different uses. Thus, a more detailed assessment of the workload from comparative risks assessments and the development of an optimal strategy for efficiently dealing with the issue would need to consider these different types of assessment situations. Such analyses will become possible as soon as the final official CFS list becomes available.

Besides the need to further clarify these details, the high number of expectable cases calls for the development of an electronic decision support instrument which would enable competent authorities to rapidly carry out initial comparative risk assessments. Such a tool should allow to filter out clear-cut cases in a semi-automatic manner and to separate them from borderline cases which require in-depth expert judgment. To this end, the legal requirement for a significant risk reduction needs to be translated into well-defined programmable decision rules. In addition, an efficient application of such a computer-based decision support tool depends on the availability of a continuously updated database with all regulatory relevant information on the risks of individual products that is required for the comparative assessments. In Germany, regulatory risk assessment reports for individual PPPs currently exist in the form of text files only, and the same may presumably apply to most other EU Member States. For performing a comparative assessment, all the relevant risk indicators, such as TER values for the various ecotoxicological endpoints, must therefore be compiled manually from the individual assessment reports. Improving this situation is essential for a less time-consuming future practice. In addition, efforts should be made to identify representative endpoints or to define suitable indicators for facilitating decision-making within the process, e.g. for sorting products according to their risk. Within a broader scope, cooperation between Member States authorities may be considered as a further means for efficiently dealing with the large numbers of comparative risk assessments needed. Comparative risk assessment of PPPs is clearly a national task, but a zonal authorisation of PPPs across different Member States has been introduced with the new PPP Regulation. In this situation, sharing the workload for comparative risk assessments between Member States in the same zone seems to be a self-suggesting option that deserves further exploration.

## Conclusions

The expectable number of cases for comparative assessments of PPPs is high. In Germany, it may comprise up to a quarter of all products and half of all uses of products. This puts regulatory authorities under considerable pressure to develop appropriate strategies for efficient handling of the task.

## Methods

Data presented in this study were generated by systematically compiling and interlinking information from three different sources:

- The FCEC report to the European Commission [11] for information on CFS properties of approved active substances (approval status as of 31 January 2013)
- The EU Pesticides database [19] for allocating approved active substances to use categories
- The database on Authorised Plant Protection Products of the German Federal Office of Consumer Protection and Food Safety (BVL) [20] for information on the number, nature, active ingredients, uses, and use categories of authorised PPP in Germany (authorisation status as of May 2013)

A list of potential CFS was derived from the FCEC report by aggregating relevant information as follows:

- CFS criterion 1 (low ADI/ARfD/AOEL): Separate assessments of ADI, ARfD, and AOEL values (Tables A1, A2, and A3 of the FCEC report) were merged under the assumption that the following decision rule applies: values are considered to fulfil the criterion when they are below the 5% percentile of a use group as defined in the EU pesticides database.
- CFS criterion 2 (two PBT criteria): Separate assessments of half-life in water, sediments, and soil (Tables A5, A7, and A9 of the FCEC report) were merged for assessments of persistence (P). Assessments of bioaccumulation (B) were directly retrieved (from Table A10 of the FCEC report). Assessments of aquatic toxicity (T_AQUA_) were generated by merging separate assessments of the toxicity to fish, algae, daphnids, and other aquatic species (as provided in Tables A11, A12, A13, and A14 of the FCEC report). Assessments of human toxicity (T_HUMAN_) were obtained by merging the separate information on CMR and STOT RE classifications (provided in Tables A16, A20, A22, A24, A26, and A 27 of the FCEC report).
- CFS criterion 4 (non-active isomers): Assessments were directly abstracted (from Table A15 of the FCEC report).
- CFS criterion 6 toxic for (reproduction 1A/1B): Separate information on existing and forthcoming classifications of reproductive toxicity was merged (from Tables A22 and A23 of the FCEC report).
- CFS criterion 7 (endocrine disrupting properties): For assessments of endocrine disrupting properties according to the interim criteria laid down in Regulation (EC) No 1272/2008, i.e. substances that are classified as both carcinogenic category 2 and toxic for reproduction category 2, separate information on these two properties (in Tables A21 and A25 of the FCEC report) were combined accordingly.
- CFS criteria 3 and 5 (‘nature of critical effects’ and carcinogen 1A/1B): No substances fulfilling any of these criteria were identified in the FCEC report.

## Abbreviations

ADI: acceptable daily intake
AOEL: acceptable operator exposure level
ARfD: acute reference dose
BVL: Bundesamt für Verbraucherschutz und Lebensmittelsicherheit (German Federal Office of Consumer Protection and Food Safety)
BfR: Bundesinstitut für Risikobewertung (German Federal Institute for Risk Assessment)
CFS: candidate for substitution
CMR: carcinogenic, mutagenic or toxic for reproduction
COM: European Commission, formerly Commission of the European Communities
EC: European Community
EEC: European Economic Community
EU: European Union
EFSA: European Food Safety Authority
FCEC: Food Chain Evaluation Consortium
JKI: Julius Kühn-Institut (German Federal Research Centre for Cultivated Plants)
MS: Member States of the EU
NOEC: no observed effect concentration
PBT: persistent, bioaccumulative, and toxic
PPP: plant protection product
REACH: registration, evaluation, authorisation and restriction of chemicals
STOT RE: specific target organ toxicity - repeated exposure
TER: toxicity/exposure ratio
UBA: Umweltbundesamt (German Federal Environment Agency)
